# Effect of Putting Grip on Eye and Head Movements During the Golf Putting Stroke

**DOI:** 10.1100/tsw.2003.14

**Published:** 2003-03-24

**Authors:** George K. Hung

**Affiliations:** Department of Biomedical Engineering, Rutgers University, Piscataway, NJ, USA

**Keywords:** eye movements, head movements, fixation, golf, putt, putting grip, conventional, cross-hand, one-handed, vestibulo-ocular response

## Abstract

The objective of this article is to determine the effect of three different putting grips (conventional, cross-hand, and one-handed) on variations in eye and head movements during the putting stroke. Seven volunteer novice players, ranging in age from 21 to 22 years, participated in the study. During each experimental session, the subject stood on a specially designed platform covered with artificial turf and putted golf balls towards a standard golf hole. The three different types of grips were tested at two distances: 3 and 9 ft. For each condition, 20 putts were attempted. For each putt, data were recorded over a 3-s interval at a sampling rate of 100 Hz. Eye movements were recorded using a helmet-mounted eye movement monitor. Head rotation about an imaginary axis through the top of the head and its center-of-rotation was measured by means of a potentiometer mounted on a fixed frame and coupled to the helmet. Putter-head motion was measured using a linear array of infrared phototransistors embedded in the platform. The standard deviation (STD, relative to the initial level) was calculated for eye and head movements over the duration of the putt (i.e., from the beginning of the backstroke, through the forward stroke, to impact). The averaged STD for the attempted putts was calculated for each subject. Then, the averaged STDs and other data for the seven subjects were statistically compared across the three grip conditions. The STD of eye movements were greater (p < 0.1) for conventional than cross-hand (9 ft) and one-handed (3 and 9 ft) grips. Also, the STD of head movements were greater (p < 0.1; 3 ft) for conventional than cross-hand and one-handed grips. Vestibulo-ocular responses associated with head rotations could be observed in many 9 ft and some 3 ft putts. The duration of the putt was significantly longer (p < 0.05; 3 and 9 ft) for the one-handed than conventional and cross-hand grips. Finally, performance, or percentage putts made, was significantly better (p <0.05; 9 ft) for cross-hand than conventional grip. The smaller variations, both in eye movements during longer putts and head movements during shorter putts, using cross-hand and one-handed grips may explain why some golfers, based on their playing experience, prefer these over the conventional grip. Also, the longer duration for the one-handed grip, which improves tempo, may explain why some senior players prefer the long-shaft (effectively one-handed grip) putter.

### INTRODUCTION

Sports medicine is becoming a very popular means of addressing specific questions posed by athletes concerning the body's forces and actions during athletic motions. It has, for example, provided valuable information about the golf swing and physical forces impacting on the golf ball. Much of this information has been obtained using high-speed photography[1] and videotape. Indeed, the components of the golf swing have been studied in great detail over past 50 years. However, there is a surprising lack of objective simultaneous measurements of eye and head motion during the golf swing, especially in putting.

Putting is a crucial element in golf. This is demonstrated by statistics compiled by the Professional Golfers Association, which showed that the best players in the world expend approximately 40% of their total strokes in a round on putting[2]. Golf teaching professionals and sports psychologists specific to golf have long taught the importance of minimal or no eye and head movements throughout the putting stroke. The eyes are important because they provide accurate perception of ball location to result in successful execution of a putting stroke. If eyes are fixated elsewhere at a position other than the ball, this can lead to an improper stroke and a missed putt. Head position is also important because it allows for maintenance of stability of the visual environment. If the head moves during the stroke, this can lead to misalignment and a missed putt.

Different putter grip styles can be seen in the golf course. Through empirical experimentation or having been taught by particular school of thought, golfers have adopted different putting grips. Indeed, many golfers try different grips over the years, and sometimes even in the course of a round. Yet there has not been any quantitative explanation as to why a certain grip works for a particular individual or under a particular circumstance (e.g., a short vs. a long putt). There are basically three types of putting grips: conventional, cross-hand, and one-handed[3,4,5]. One-handed putting represents the grip used for the long-shafted putter, where the left hand hold the putter near the chin or chest and is anchored there. A detailed description of these grips is given in the Methods section. Also, for this article, only the right-handed putting style will be considered, although the results can be generalized for the left-handed player.

To quantitatively assess the effect of grip style on eye and head movements, the eye, head, and putter positions were recorded simultaneously during a putting stroke using the three putting-grip styles. Seven novice players served as subjects. These players were chosen in part because they possessed less preconceived bias in their putting-grip style than more experienced players. Nevertheless, the findings should be applicable to golfers regardless of their level of expertise. The results show differences in eye and head movements for the different putting-grip styles, which may provide important insights into the underlying mechanisms behind the selection of particular putting-grip styles by individual golfers.

## METHODS

### Apparatus

The laboratory environment was designed to present, as closely as possible, a real-life putting green environment while allowing for the recording of data. It consisted of a putting platform, putter position sensor, helmet-mounted eye movement sensors, and head movement measurement device (Patent pending: serial nos. 60/296,527 and 60/317,944 [6]; see Fig. 1A, B). The putting platform is covered by green artificial turf that mimics the texture of the putting green. The platform is constructed of standard 5/8-in.-thick particleboard, stacked three-boards thick to provide the full thickness of the platform. The platform is comprised of three sections. The first section, where the subject would stand, is 3 × 3 ft. The second section is 3 × 2 1/2 ft, whereas the third section is 6 × 2 1/2 ft. The width of sections two and three are set at 2 1/2 ft instead of 3 ft to conform to laboratory space constraints. Also, to reduce the weight and cost of these two sections, runners 2 1/2 ft long × 2 in. wide × 1 1/4 in. high (i.e., 2 boards' thickness) are placed under the top platform board at approximately every 3 ft. The starting ball position is located 2 ft from the edge of section one, whereas the golf hole having a standard 4 3/16 in. diameter is located 1 ft from one end of the 6-ft-long third section. Thus, by adjustment of the positions of the sections, putting distances of either 3 or 9 ft are possible.

The putting position sensor consists of 16 infrared phototransistors place in the middle-level particleboard in the first section (Fig. 2A, B). They are inserted into different pin positions in three electronic breadboards (6 1/2 in. long × 2 1/4 in. wide × 5/16 in. thick), which are sandwiched and secured in place by particleboards. The resolution of the putting sensor is provided by the minimum spacing (1.2 cm) between phototransistors. The breadboards, and in turn the phototransistors, are aligned in the direction of the putting stroke. A ribbon cable connected to the phototransistors transfers the electronic signals to a circuit board outside of the platform. The circuit board processes the electronic signals to provide a digital signal representing the instantaneous position of the putter face. The signal is input to a PC.

The binocular horizontal eye movement monitor is a Skalar-Iris (Model 6500) helmet-mounted infrared reflection device (Fig. 3). It has a linear range of ±25º, a resolution of 5 min of arc, and a bandwidth of 200 Hz. The voltage output signals representing eye rotations in the two eyes are input to a PC.

The head rotation movement sensor consists of a potentiometer that is placed in an adjustable mount above the subject's head (Figs. 4A, B). (In our previous experiments, we found that head translation movements were either small or negligible during the putting stroke). A small rectangular magnet is glued to the end of the potentiometer and is bounded by two plastic rectangular pieces to form the female coupling member (Fig. 4A). Similarly, a small rectangular magnet is screwed into a cylindrical unit, which itself is screwed onto the top the helmet, to form the male coupling member (Fig. 4B). At the beginning of an experimental session, while the subject takes his/her normal putting stance, the position of the head movement sensor unit is adjusted until the male and female components are joined and the axis of the potentiometer stem is aligned approximately with an imaginary axis through the top of the head and its center-of-rotation (Fig. 5). Rotation of the head about this axis will result in rotation of the potentiometer, which in turn causes a change in voltage in the head sensor output signal. The head movement sensor has a resolution of 0.5 cm and is linear throughout the ±25.0 cm display range (see below for conversion from head angular rotation in degrees to equivalent platform displacement in centimeters). The voltage signal, which represents the head position, is then input to the PC. The head movement sensor can be easily disengaged from the helmet by simply having the subject

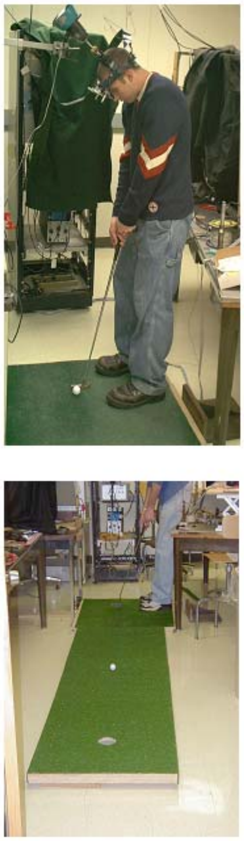

(A)

(B)

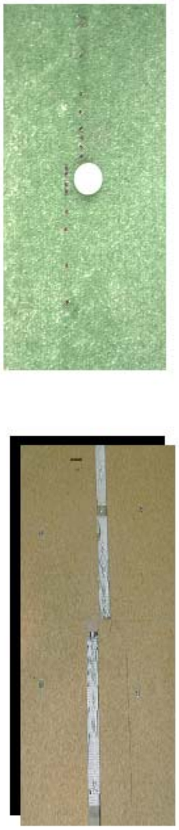

**FIGURE 1**. (Left column of figures) Putter platform in use during an experimental trial. (A) Subject wearing helmet (for eye and head movement measurements) and preparing to putt on the first section of the platform. Note that under this section are the embedded photodetector arrays (see Fig. 2). (B) Two other sections are combined with the first section to provide the setup for the 9-ft putts. Turning around the third section (where the hole is) and removing the middle section allows for 3-ft putts.

**FIGURE 2**. (Right column of figures) Top view of first section of platform where the subject stands (see Fig. 1A) showing ball and photodetector locations. (B) Arrays of photodetectors embedded under the artificial turf in the platform. Note the front array is offset so that the ball movement will not trigger the detectors. The relative position of the golf hole is in front of the photodetectors (i.e., in the direction below the figure).

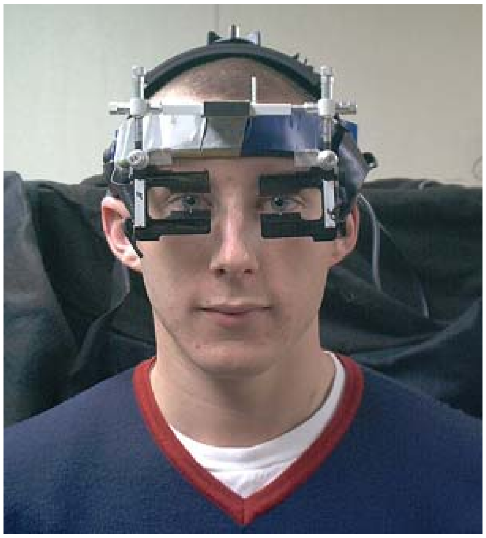

**FIGURE 3**. Subject wearing helmet containing the binocular eye movement sensor unit. Eye movement sensor unit in each eye contains arrays of infrared emitters (upper black rectangular piece) and detectors (lower black rectangular piece). As the eye rotates, the amount of infrared reflection from the white part of the eye changes, which can be recorded as a voltage change.

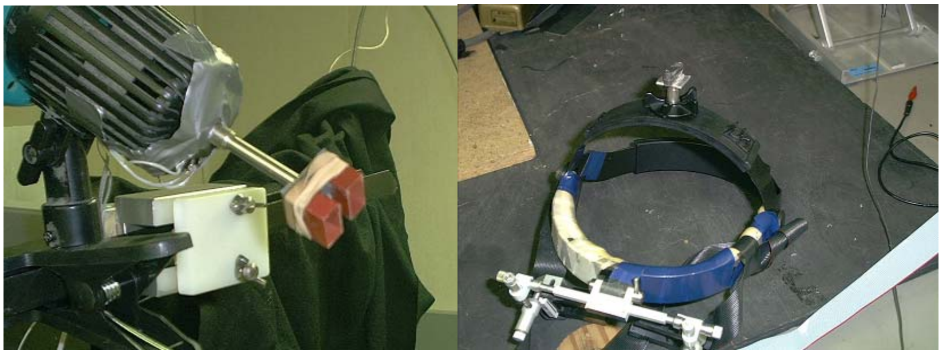

**FIGURE 4**. (A) Female component of the head movement sensor consisting of a potentiometer cemented to a swivel unit (top left). A small square aluminum platform is cemented to the end of the potentiometer shaft. The rectangular magnet is cemented on top of the platform, and is flanked by two rectangular plastic members. (B) Male component of the head movement sensor, consisting of a rectangular magnet mounted on top of a threaded cylindrical unit, which in turn is screwed onto the top portion of the eye movement helmet.

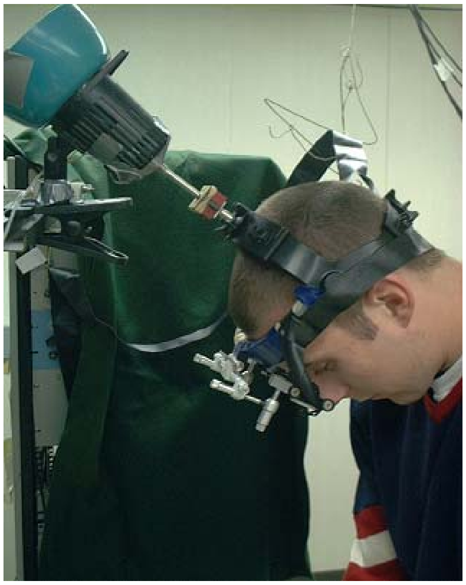

**FIGURE 5**. The head movement sensor is assembled by attaching the female component in the head sensor swivel unit (see Fig. 4A) to the male component in the helmet (see Fig. 4B). The swivel unit is clamped onto a frame with three directions of freedom of movement. The swivel unit itself can be pivoted and rotated about its stem. This allows for different subject heights and head angles during the putting stroke. During the experimental setup, the shaft of the potentiometer that is attached to the swivel unit (see Fig. 4A) is aligned with the head in such a way that only rotation about an imaginary axis through the top of the head and its center-of-rotation will turn the potentiometer, which in turn provides the head rotation signal.

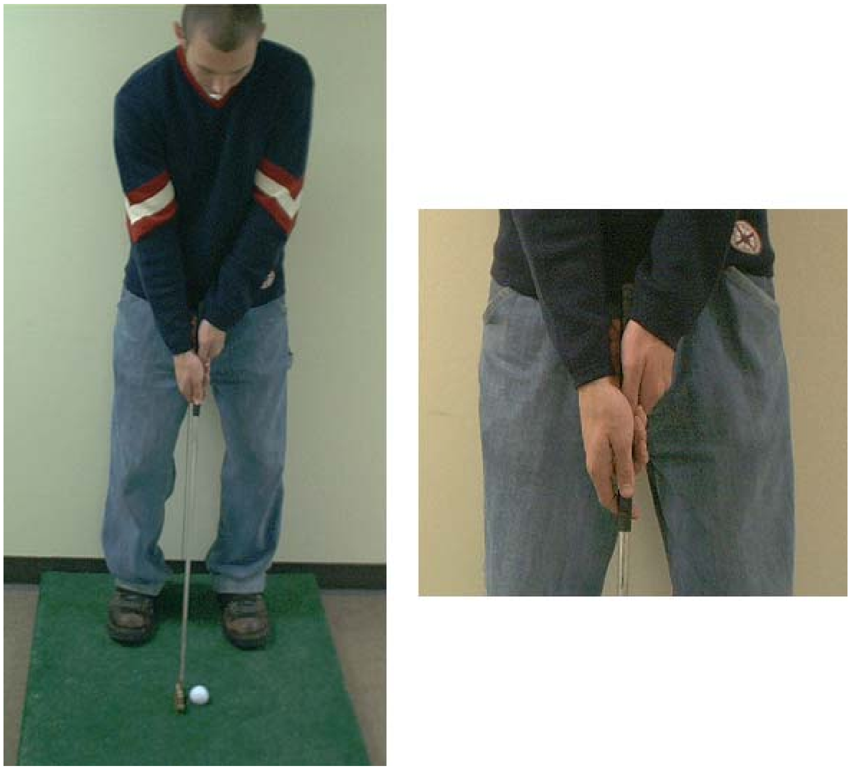

**FIGURE 6**. (A) Overall view of conventional grip for putting in a novice player (GH), where the right hand is *below* the left hand. (B) Close-up view of the conventional grip.

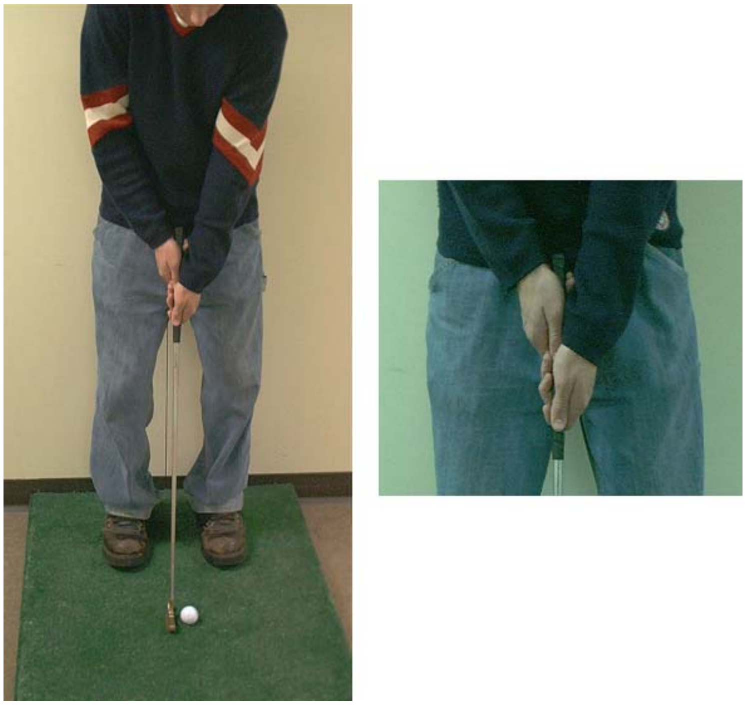

**FIGURE 7**. (A) Overall view of cross-hand grip for putting in a novice player (GH), where the right hand is *above* the left hand. (B) Close-up view of the cross-hand grip.

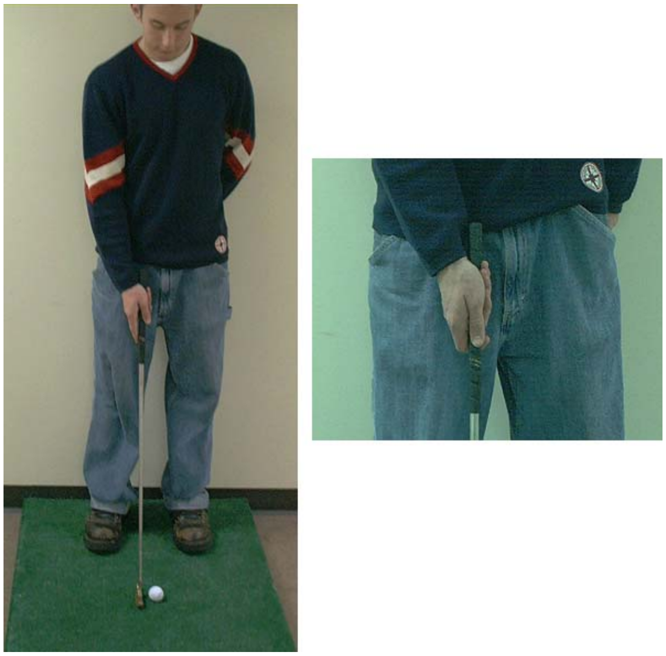

**FIGURE 8**. (A) Overall view of one-handed grip for putting in a novice player (GH), where the left hand is placed behind the subject's back. (B) Close-up view of the one-handed grip.

take a step back away from the device. A more recent innovation in our laboratory is the use of an accelerometer to measure head acceleration, then wirelessly transmit this signal to a receiver, and electronically integrated twice to obtain head rotation position signal[6].

#### *Putting-Grip Styles*

Three putting-grip styles were used. In the conventional grip (Fig. 6A, B), the left hand holds the top of the grip portion of the putter shaft while the right hand is placed below the left. In most players, the little finger of the right hand is placed in a position just overlapping the gap between index and middle fingers of the left hand. In the cross-hand grip (Fig. 7A, B), the positions of the hands are reversed. The right hand holds the top of the grip portion of the putter shaft while the left hand is placed below the right. In this position, the tip of the index finger of the right hand may be in contact with the wrist of the left hand. In the one-handed grip (Fig. 8A, B), which represents the action used for the long-shafted putter, the right hand holds approximately the middle portion of the putter grip. For consistency in interpreting the results, the same putter (37 in. Acushnet Bulls Eye FLU5S) was used for all three putting-grip styles in all subjects.

### Experimental Procedure

Seven unpaid volunteer subjects were recruited from the Rutgers University student community via word-of-mouth and advertisement. They were all novice right-handed golfers ranging in age from 21 to 22 years, with corrected vision of 20/20 or better. All were male except for one female. Informed consent was obtained prior to experimentation. The research followed the tenets of the Declaration of Helsinki and the protocol was approved by the Rutgers Institutional Review Board.

The experimental paradigm for each subject was as follows: the subject first practiced five putts without the helmet to become familiar with the conditions. Also, for each of the three putting-grip styles, as the subject took a putting stance, the distance from the ball on the platform to the subject's eyes was measured. Just prior to the beginning of the experiment, the helmet was placed on the subject's head, and the positions of the infrared detectors for the two eyes were properly adjusted. At the beginning of the trials, an eye movement calibration trial was performed, where the subject fixated sequentially on three known locations (left end, middle, and right end of the phototransistor array) on the platform. This provided accurate recording of eye fixations during a putting stroke, measured in units of centimeters of displacement on the platform. For the putting trials, the head movement sensor was attached to the head. The potentiometer was precalibrated to provide the conversion from a measured voltage change to the corresponding angular rotation of the potentiometer shaft, which in turn corresponded to the angular rotation of the head. This angle was converted to the displacement of a hypothetical beam emanating from the center-of-rotation of the head (approximated by the position of the center between the two eyes) on the platform, in centimeters. In this way, all measurements (i.e., putter, eye, and head movements) had the consistent unit of centimeters of platform displacement. The subject was instructed to attempt to putt the ball into the hole using as natural a stroke as possible. Each putt constituted a trial lasting 3 s, and the result of the putt (i.e., where the putt ended up relative to the hole) was tabulated in a data sheet. For each of two distances (3 and 9 ft), 20 trials were performed and repeated for each of the three putting-grip styles. The entire experiment for each subject required about 90 min.

For each 3-s trial, the putter, left eye, right eye, and head position time courses were recorded on a PC at a sampling rate of 100 Hz. The resulting data were analyzed using programs written in C++ and MATLAB codes. The results were displayed in four channels as position time courses for putter, left and right eye movements, and head movement. Also displayed were the corresponding velocity traces. For each record, the beginning of the putt as well as the end of the putt (i.e., point of impact, or at the point corresponding to the return to the initial putter position) were marked visually. The data were analyzed to provide the duration of the putt and the standard deviations (STD; i.e., relative to the initial position) of combined left and right eye movements as well as head movements during the putt (i.e., from the beginning of the backstroke to the point of impact).

The data from each subject were averaged, and the averaged data for the seven subjects were used in the statistical analysis. One-tailed t-tests (MATLAB Statistical Analysis Toolbox) were performed to assess the statistical significance of differences between the different putting-grip styles for the various parameters: height to eyes, putt amplitude, duration of putt, percentage of putts made, STD of combined left and right eye movements during the putt, and STD of head movements during the putt.

### RESULTS

#### Typical Movements

Typical 9-ft putt time courses for the three putter-grip styles are shown in Figs. 9A-C to illustrate the general pattern of responses. The sign convention is as follows: the forward stroke of the putt, leftward eye movement, and leftward head rotation (i.e., nose points leftward towards the hole) are assigned positive values. For the conventional-grip putt (Fig. 9A), note the slight rightward rotation of the head (i.e., negative value, or away from the hole direction), probably due to shoulder rotation, during the backstroke, and simultaneous automatic compensatory smooth eye movements (called vestibulo-ocular response [VOR], or compensatory rotation of the eyes in a direction opposite to head rotation to maintain stable fixation in space[7]) in the positive (or towards the hole) direction. These two movements generally tend to cancel each other to provide relative steady eye fixation on the ball. However, incomplete compensation or disruptions due to other eye or body motions will result in inappropriate eye fixation in space. Note also that at about the moment of impact, there is a saccadic eye movement (or rapid rotation of the eyes to acquire a new target, such as that seen during reading[8,9,10,11,12]) in the direction of the hole, which may possibly affect the release of the putter following impact. The putting stroke shows relatively good positive acceleration in the forward stroke, reaching approximately peak velocity at impact (0 cm putter position).

For the cross-hand grip putt (Fig. 9B), note the slight rightward head rotation away from the hole, along with a slight compensatory VOR during the putt. There is also a saccade in the direction of the hole that occurs prior to impact, but the overall eye position is approximately directly on the ball at the moment of impact. Subsequent saccades and head rotations are simply tracking movement in following the path of the ball towards the hole. Moreover, the putting stroke is near peak velocity at the moment of impact.

For the one-handed grip putt (Fig. 9C), note the near absence of head movements during the stroke, and the eyes are fixated approximately on the ball throughout the stroke. Also, the putting stroke is near peak velocity at the moment of impact. Subsequent saccadic and head rotations are associated with viewing the path of the ball towards the hole. Also note that the putter is held for a relatively prolonged period during the backstroke.

### General Findings

The results show differences between putting-grip styles [conventional (Conv); cross-hand (Cross); one-handed (One)] for various parameters: height to eyes, putt amplitude, putt duration, percentage of putts made, STD left and right eye movements during the putt, STD of head movements during the putt (Table 1). A simpler-to-interpret illustration of the same information as in Table 1 is shown in Table 2.

The data in Table 1 can be interpreted using an example. For the first two columns under "Height to Eyes" for the 3-ft putt, the mean for the conventional and cross-hand grips are 142.6 and 139.3 cm. To determine whether the difference between the means is significant (i.e., is conv > cross), a one-tailed t-test was applied (first column). The result is that $p = 0.031$, indicating the difference is significant (i.e., $p < 0.05$). A similar interpretation is used for all the other columns.

"Height to Eyes" is highest for one-handed, intermediate for conventional, and lowest for cross-handed. This is because for the one-handed putt, the subjects tend to stand more erect. On the other hand, for the cross-hand grip, the left hand is positioned lower on the putter grip, thus lowering the left shoulder. This tends to bring the head down as well, thus lowering the height of the head relative to the platform. The "Height to Eyes" findings are obviously the same for 3- and 9-ft putts. They are shown for both distances in Table 1 for consistency in appearance of the two subtables.

"Putt Amplitude" is not significantly different for the three putting-grips style for the 3-ft putt. However, for the 9-ft putt, amplitude for the cross-hand grip is statistically significantly smaller than either conventional or one-handed grip. This may be due to a restriction of the right elbow motion in the backstroke as a result of the increased bend of the right elbow to compensate for the higher right hand position on the putter for the cross-hand grip. "Putt Duration" is longer for the one-handed than either conventional or cross-hand grip, for both 3- and 9-ft putts. This may be due to the increased length of the swing arm (putter plus hand and arm) in the one-handed putt. Hence, if one considers this as a pendulum motion, the increased length corresponds to an increase in the period of motion, and in turn an increase in the duration of the putt.

"Percent Made" appears to be higher for cross-hand than either conventional or one-handed grip, for both 3- and 9-ft putts (see Table 1, mean values), but these comparisons are not statistically significant except for one condition. For the 9-ft putt, the "Percent Made" is statistically-significantly higher for the cross-hand than conventional grip ($p = 0.006$).

The "STD of Combined Right and Left Eye Movements" is lowest for one-handed, intermediate for cross-hand, and highest for conventional, for both 3- and 9-ft putts. This holds except for the comparison between cross-hand and conventional for the 3-ft putts ($p = 0.312$).

The "STD of Head Movements" is lowest for one-handed, intermediate for cross-hand, and highest for conventional, for the 3-ft putts. This holds except for the comparison between one-handed and cross-hand putts ($p = 0.151$). However, for the 9-ft putts, there are no statistically significant differences among the putting-grip styles. These findings suggest that head motion plays a more important role in shorter (3 ft) than longer (9 ft) putts, and moreover indicates that one-handed grip, and to a lesser extent the cross-hand grip, result in less head motion during the putt.

**TABLE 1**
**Comparisons Among Conventional (Conv), Cross-Hand (Cross), and One-Handed (One) Grips for the Averaged Data in Seven Subjects Using the t-Test (One Tail, Significance Level Set at *p* = 0.05) for 3- and 9-ft Putts**

| | **3-ft Putts** | | | | | | | | |
|---|---|---|---|---|---|---|---|---|---|
| | **Height to Eyes** | | | **Putt Amplitude** | | | **Putt Duration (s)** | | |
| Mean | Conv 142.6 | Cross 139.3 | One 145.1 | Conv 16.80 | Cross 16.64 | One 17.04 | Conv 0.79 | Cross 0.75 | One 0.87 |
| Hypothesis | Conv > Cross | Cross < One | One > Conv | Conv > Cross | Cross < One | One > Conv | Conv > Cross | Cross < One | One > Conv |
| t - test | *p* = 0.031 SG | *p* = 0.002 SG | *p* = 0.032 SG | *p* = 0.417 NS | *p* = 0.334 NS | *p* = 0.362 NS | *p* = 0.213 NS | *p* = 0.005 SG | *p* = 0.004 SG |
| | **Percent Made (%)** | | | **STD LE + RE Movement** | | | **STD Head Movement** | | |
| Mean | Conv 71.20 | Cross 78.14 | One 71.09 | Conv 4.41 | Cross 4.15 | One 3.30 | Conv 2.75 | Cross 2.09 | One 1.65 |
| Hypothesis | Conv < Cross | Cross > One | One < Conv | Conv > Cross | Cross > One | One < Conv | Conv > Cross | Cross > One | One < Conv |
| t - test | *p* = 0.121 NS | *p* = 0.121 NS | *p* = 0.493 NS | *p* = 0.312 NS | *p* = 0.066 TN | *p* = 0.025 SG | *p* = 0.076 TN | *p* = 0.151 NS | *p* = 0.018 SG |
| | **9-ft Putts** | | | | | | | | |
| | **Height to Eyes** | | | **Putt Amplitude** | | | **Putt Duration (s)** | | |
| Mean | Conv 142.6 | Cross 139.3 | One 145.1 | Conv 22.13 | Cross 20.38 | One 22.55 | Conv 0.77 | Cross 0.80 | One 0.91 |
| Hypothesis | Conv > Cross | Cross < One | One > Conv | Conv > Cross | Cross < One | One > Conv | Conv < Cross | Cross < One | One > Conv |
| t - test | *p* = 0.031 SG | *p* = 0.002 SG | *p* = 0.032 SG | *p* = 0.052 TN | *p* = 0.016 SG | *p* = 0.369 NS | *p* = 0.300 NS | *p* = 0.001 SG | *p* = 0.001 SG |
| | **Percent Made (%)** | | | **STD LE + RE Movement** | | | **STD Head Movement** | | |
| Mean | Conv 33.4 | Cross 47.9 | One 41.6 | Conv 5.91 | Cross 5.06 | One 4.23 | Conv 2.79 | Cross 3.04 | One 2.16 |
| Hypothesis | Conv < Cross | Cross > One | One > Conv | Conv > Cross | Cross > One | One < Conv | Conv < Cross | Cross > One | One < Conv |
| t - test | *p* = 0.006 SG | *p* = 0.274 NS | *p* = 0.242 NS | *p* = 0.099 TN | *p* = 0.016 SG | *p* = 0.038 SG | *p* = 0.358 NS | *p* = 0.153 NS | *p* = 0.127 NS |

STD = standard deviation; SG = significant, $p < 0.05$; TN = trend, $0.05 \leq p \leq 0.10$; NS = not significant. All values in centimeters of platform displacement except where indicated.

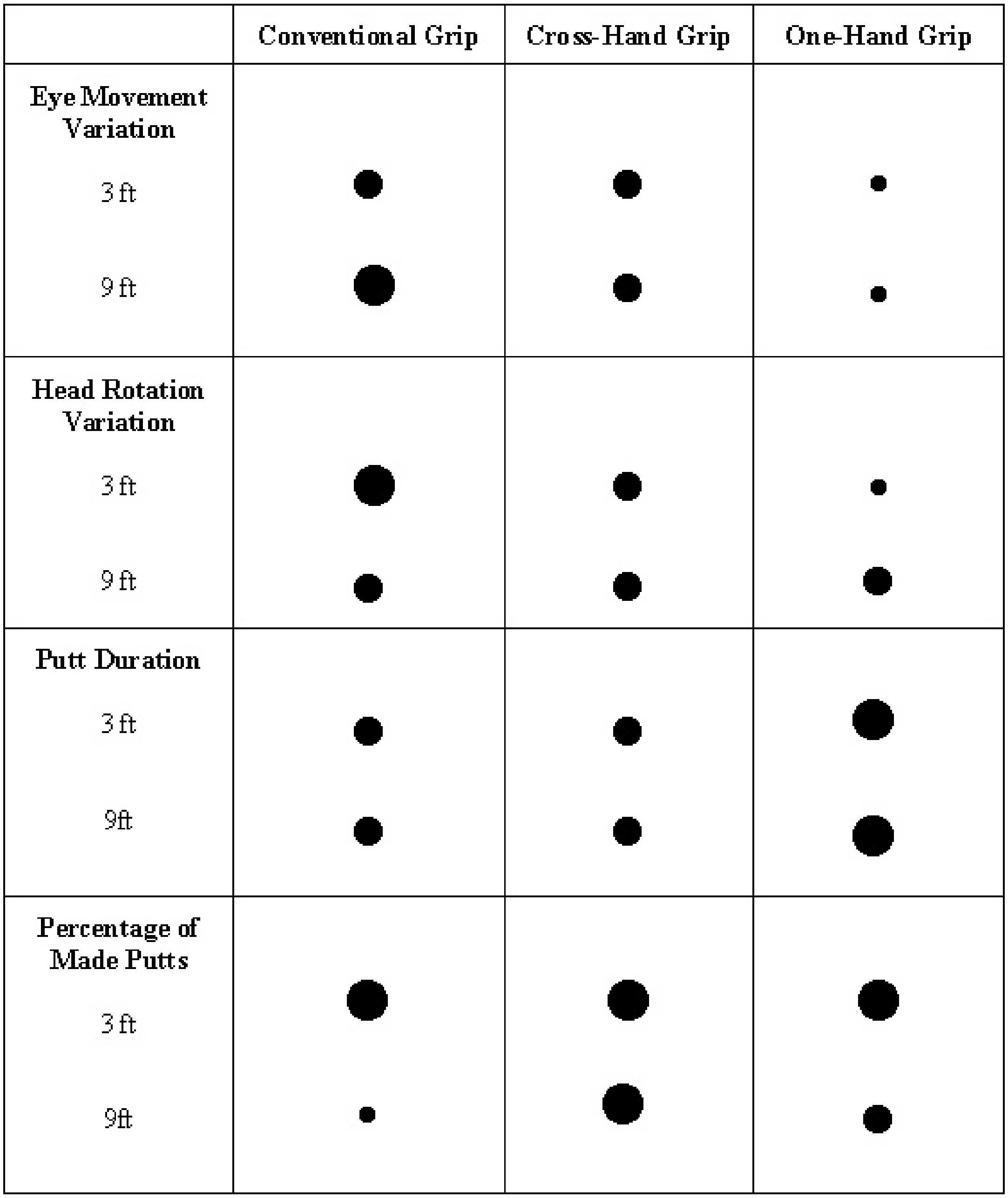

*Note: Circle size represents relative parameter magnitude.

**Table 2.** Simpler-to-interpret illustration of same information as in Table 1.

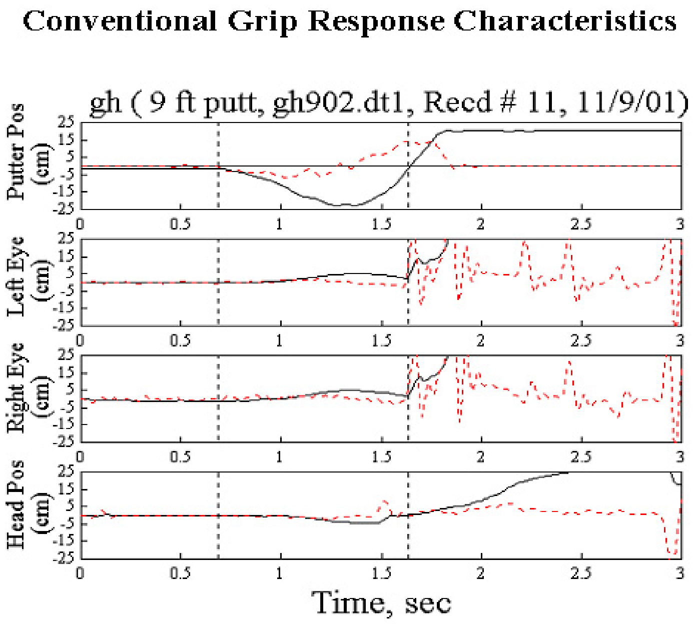

**Figure 9A.** Time traces of putter, eye, and head movements for a typical 9-ft putt using the conventional grip. The hole location is in the direction of the top of the page. Positive and negative numbers represent movements towards or away from the hole, respectively. Top trace - Putter position. 2nd and 3rd traces - Left and right eye positions, respectively. The eye movements, via the calibration procedure, are in the unit of cm displacement on the platform. Bottom trace - Head rotation. The angle of rotation about an imaginary axis through the top of the head and its center-of-rotation has been converted to equivalent movement of an imaginary beam projected from the center-of-rotation of the head (approximated by the center position between the two eyes) onto the platform, in cm. For all traces, the position and velocity are designated by solid and dashed lines, respectively. Vertical dashed lines were visually selected to encompass the duration of the putt from the beginning of the backstroke to the point of impact. Sub. GH.

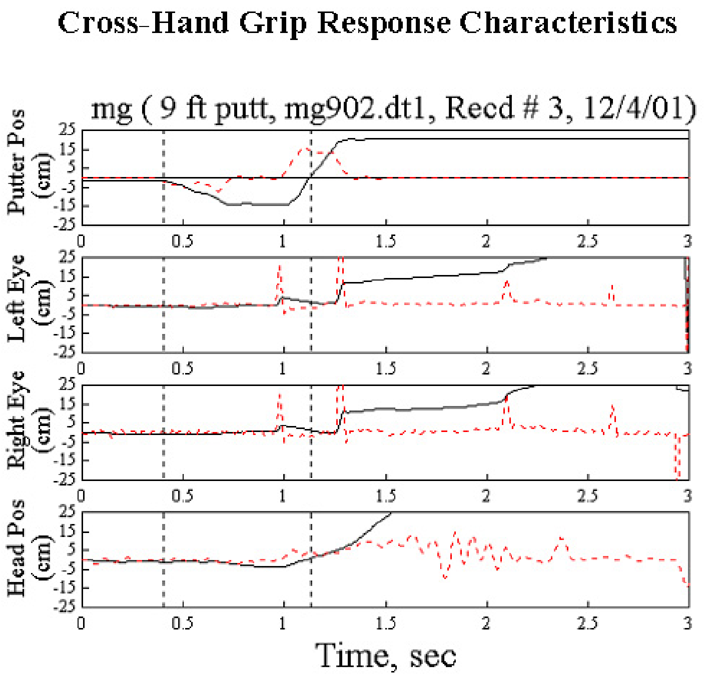

**Figure 9B.** Time traces for a typical 9-ft putt using the cross-hand grip. Sub. MG.

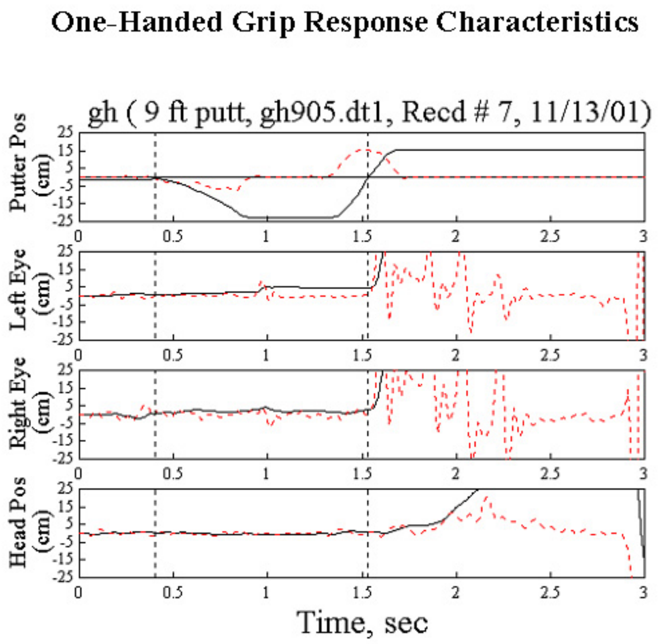

**Figure 9C**. Time traces for a typical 9-ft putt using the one-handed grip. Sub. GH.

### DISCUSSION

This study has provided some important quantitative insights into the effect of grip style on eye and head movements during the putting stroke. For minimizing head movements, there appears to be a slight advantage of the one-handed grip over the other two grip styles, especially for the shorter putts. This may be because for the conventional and cross-hand grips, the right and left shoulders are linked due the coupling of the two hands. Thus, during the backstroke, the movement of the hands causes the left shoulder to dip slightly and the right shoulder to rise slightly. The natural linkage of the shoulders to the head causes it to rotate slightly clockwise, as seen from above. The opposite occurs during the forward stroke. In contrast, for the one-handed grip, the two hands are not linked, so that movements of the right hand and arm during the putting stroke rotate the right shoulder, but with relatively little dip or rising motion. Moreover, since there is less constrained coupling of the right shoulder to the head, the head motion is relatively small during the putting stroke. Generally, players are reluctant to attempt the one-handed putt perhaps because of a feeling that they may lose control of the stroke. However, some professional players have used the one-handed putting grip, notably Mike Hulbert, who used it in the mid-1990s, and Lanny Watkins, who used it for short putts. Nowadays, the one-handed putting style is manifested in the use of the long-shafted putter, which is quite popular among senior players. An additional reason for its use among senior players is discussed below.

Variation in eye movements were found to be least for one-handed, intermediate for cross-hand, and highest for conventional grip. This is due in part to the VOR. Thus, the smaller eye movements for one-handed grip is partly due to the smaller head movements. However, not all eye movement effects are a reflection of the VOR, since the statistical comparisons for the different grips are not identical for eye and head movements (see Table 1). There may be additional factors, such as posture and initial angle of the head relative to the ball on the platform that can affect the scanning eye movements during the putting stroke.

The percentage of putts made was higher for cross-hand than conventional grips for 9-ft putts. It may be possible that the higher percentage made for the cross-hand grip is due a slightly more "solid" impact of the putter with the ball, especially for 9-ft putts, as reported by some of the novice subjects. On the other hand, although the direction of the putt appears to be better for the cross-hand grip, there may be a reduction in control of distance of the putt. This is seen in some of the novice player's putts that "fly past the cup". Thus, there is not a clear-cut advantage of the cross-hand over the conventional grip.

The duration of putts made was longer for one-handed than the other two grip styles. This appears to be due to the greater effective length of the swing arm (putter plus hand and arm) of the putt. If one considers the putting motion as being analogous to that of a pendulum, in which the period is related to its length, then the longer swing arm for the one-hand grip would be associated with a longer duration. A longer duration or slower putt may provide improved timing in the putting stroke, thereby providing greater consistency. This may explain why many senior golfers have, through empirical experimentation with various putters, adopted the long-shafted putter (using effectively the one-handed grip). Perhaps the reduction in timing and control as one ages can be compensated in part by the use of a putting grip style that provide a slower, more controlled, tempo.

The subjects used in this study were all college students between the ages of 21 and 22. Most of them played other sports but all were novice golfers. This pool of novice young golfers has the advantage of not having any preconceived bias in favor of a particular golf grip. Also, they all have relatively good eye-hand coordination, so that this would not be a limiting factor in the study. Moreover, the narrow range of ages ensures a relatively uniform subject pool, without large age disparities. It would, however, be helpful in a follow-up study to investigate this effect in pools of subjects of various age groups. Further, it would be very informative to test this in expert golfers. Indeed, plans are under way to test members of the Rutgers University men and women golf teams.

### CONCLUSION

The experimental findings showed smaller variations using the cross-hand and one-handed grips than the conventional grip. This holds both for eye movements during longer putts and head movements during shorter putter. Also, the one-handed grip exhibited longer duration than the two other grip styles. However, when considered in terms of practical applications, each putting grip style has its advantages and disadvantages. The conventional stroke feels more natural and provides a sense of control over the putt. This must be weighed against the perhaps slightly more "solid" impact of the cross-hand grip and the better timing provided by the one-handed grip. The cross-hand grip tends to lock the left hand and arm in a relatively fixed position, and thus reduces any "conflict" between the right and left hands' neuromotor commands, which may cause the "yips" (a golf terminology indicating a psychophysical condition characterized by jitters during the putting stroke). This, however, must be weighed against the slightly reduced control over the distance of the putt. The one-handed grip provides the least amount of head and eye movements, and moreover, can be surprisingly accurate. This, however, must be weighed against the feeling of loss of control over the stroke, although this is offset in part by the use of a long-shafted putter. What do these finding tell us about the appropriate putting grip? Perhaps a combination of grips should be considered. For example, for the longer putts, use a conventional or cross-hand grip, whereas for the shorter putts, use a one-handed or cross-hand grip. Each individual must decide for oneself, through experimentation on the putting green and the golf course, which grip or combination of grips works best. This study has provided some of the quantitative and scientific rationale for gaining insights into the mechanisms underlying such a decision.

### ACKNOWLEDGEMENTS

The author thanks Rutgers University students Gary Horton, Brian Cicalese, Nicholas Frietag, and Shana Groeschler for their assistance in the laboratory.
